# GADD153 expression does not necessarily correlate with changes in culture behavior of hybridoma cells

**DOI:** 10.1186/1472-6750-7-89

**Published:** 2007-12-10

**Authors:** Matthew Mallory, Kevin Chartrand, Eric R Gauthier

**Affiliations:** 1 Department of Biology, Laurentian University, Sudbury, Ontario, Canada; 2 Department of Chemistry and Biochemistry, Laurentian University, Sudbury, Ontario, Canada

## Abstract

**Background:**

The acute sensitivity of some hybridoma cell lines to culture-related stresses severely limits their productivity. Recent developments in the characterization of the stress signals modulating the cellular phenotype revealed that the pro-apoptotic transcription factor Gadd153 could be used as a marker to facilitate the optimization of mammalian cell cultures. In this report, we analyzed the expression of Gadd153 in Sp2/0-Ag14 murine hybridoma cells grown in stationary batch culture and subjected to two different culture optimization paradigms: L-glutamine supplementation and ectopic expression of Bcl-xL, an anti-apoptotic gene.

**Results:**

The expression of Gadd153 was found to increase in Sp2/0-Ag14 cells in a manner which coincided with the decline in cell viability. L-glutamine supplementation prolonged Sp2/0-Ag14 cell survival and greatly suppressed Gadd153 expression both at the mRNA and protein level. However, Gadd153 levels remained low after L-glutamine supplementation even as cell viability declined. Bcl-xL overexpression also extended Sp2/0-Ag14 cell viability, initially delayed the induction of Gadd153, but did not prevent the increase in Gadd153 protein levels during the later phase of the culture, when cell viability was declining. Interestingly, L-glutamine supplementation prevented Gadd153 up-regulation in cells ectopically expressing Bcl-xL, but had no effect on cell viability.

**Conclusion:**

This study highlights important limitations to the use of Gadd153 as an indicator of cell stress in hybridoma cells.

## Background

Mammalian cell lines provide several advantages over other cellular systems for the production of recombinant proteins, most notably the correct processing and modification of mammalian proteins [1]. Unfortunately, several mammalian cell lines undergo apoptotic death upon exposure to stresses originating from large scale cultures (nutrient starvation, hypoxia, shear stress, osmotic stress), severely limiting their productivity [2]. Therefore, substantial efforts have been made in the past few years to devise strategies that reduce the loss of cell viability and increase the productive life of the cells. This includes 1) culture supplementation with limiting nutrients [3] and 2) cellular engineering by transfecting cell lines with cDNA molecules encoding anti-apoptotic proteins (e.g. Bcl-2 family proteins) [4].

Recent efforts have also focused on the characterization of the stress-induced signaling pathways leading to changes in the cellular phenotype [5,6]. Of particular interest is the identification of stress-related markers that would facilitate the optimization of mammalian cell culture processes. One such promising marker is Gadd153, a basic domain-leucine zipper (bZip) transcription factor of the C/EBP family [7]. A pro-apoptotic protein, Gadd153 has been shown to be up-regulated by several stresses found in large scale cultures, such as amino acid or glucose starvation [8,9], endoplasmic reticulum stress [10], osmotic stress [11] and hypoxia [12]. Gadd153 mRNA and protein levels are increased during the decline phase of NS0 [13] and CHO cultures [14], and nutrient supplementation is sufficient to decrease Gadd153 expression and improve cell survival in batch culture [13,14]. While its involvement in the induction of apoptosis in NS0 cultures has recently been disputed [15], the tight regulation of Gadd153 by culture-related stresses makes it a promising indicator of culture health.

The mouse hybridoma Sp2/0-Ag14 (Sp2/0) cell line offers several advantages for studying the regulation of hybridoma cell viability. Firstly, this cell line is acutely sensitive to culture-related stresses. In particular, Sp2/0 cells rapidly undergo apoptosis after 4 days of stationary batch culture due to L-glutamine depletion [16]. Thus, the viability of Sp2/0 cells can be greatly improved by L-glutamine supplementation on culture day 4 [16]. Secondly, Sp2/0 cells express low levels of the anti-apoptotic proteins Bcl-xL and Bcl-2 [17]. This makes this cell line readily amenable to cellular engineering experiments involving the ectopic expression of these anti-apoptotic proteins [16,18].

In this report, we studied the regulation of Gadd153 in wild type Sp2/0 cells and in Sp2/0 cells overexpressing Bcl-xL. Our results show that, while Gadd153 is tightly regulated by L-glutamine in both cell lines, Gadd153 protein levels remained low after L-glutamine supplementation, even as cell viability progressively decreased. Furthermore, reducing Gadd153 levels by L-glutamine supplementation in the Bcl-xL-expressing cell line did not impact on cell viability. This indicates that the usefulness of Gadd153 as a marker of the health of mammalian cell cultures is limited.

## Results

### Gadd153 is induced in declining Sp2/0 cultures

**Figure 1:**
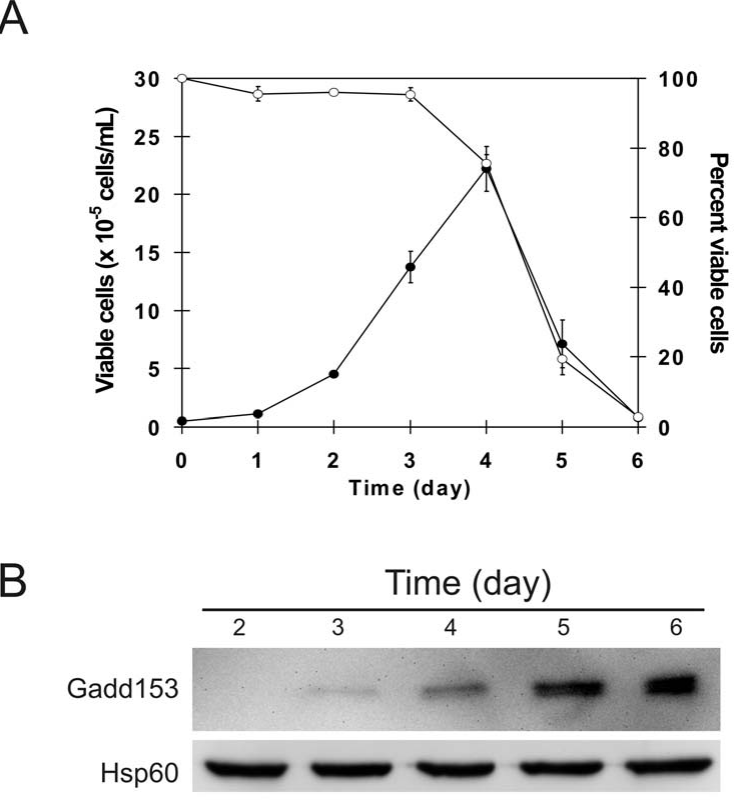
**Expression of Gadd153 in Sp2/0 stationary batch cultures**. A) Stationary batch culture of Sp2/0 cells. Closed circles: viable cell density. Open circles: Percentage of viable cells in the culture. Data are expressed as the average ± standard deviation of 3 independent experiments. B) Immunoblot analysis of Gadd153 expression during batch culture (data representative of 3 independent experiments).

We first analyzed the regulation of Gadd153 protein levels in Sp2/0 cells grown under stationary batch culture conditions. Gadd153 expression was very low during the first 4 days of culture, which corresponded to the lag and exponential phases of cell growth (Fig. 1A and 1B). However, Gadd153 protein levels increased sharply on culture day 5, correlating with a precipitous decline in cell viability. These data indicate that, because of the acute increase in its expression in the declining phase of Sp2/0 cultures, Gadd153 might be used as an indicator to monitor the health of growing Sp2/0 cells.

### Modulation of Gadd153 expression by L-glutamine supplementation

**Figure 2:**
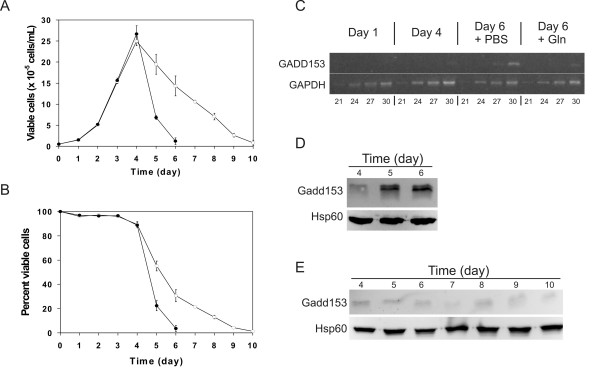
**Effect of L-glutamine supplementation on the expression of Gadd153**. Sp2/0 cells were processed for batch culture and supplemented on culture day 4 with either PBS (closed symbols) or L-glutamine (final concentration: 4 mM. Open symbols.). A) Viable cell density. B) Percentage of viable cells in the culture. C) Semi-quantitative RT-PCR analysis of Gadd153 mRNA levels. Numbers at the bottom of the figure indicate the number of amplification cycles. D) Immunoblot analysis of Gadd153 expression in the PBS-treated control. E) Immunoblot analysis of Gadd153 expression in the L-glutamine-supplemented culture. Data in panels A and B are expressed as the average ± standard deviation of 3 independent experiments. Data in panels C-E are representative of 3 independent experiments.

For Gadd153 to be useful to monitor the stress level of hybridoma cultures, its expression must be responsive to strategies that improve cell viability. Therefore, we investigated the impact of L-glutamine supplementation on the expression of Gadd153 in Sp2/0 stationary batch cultures. In agreement with our previous observations [16], L-glutamine supplementation on culture day 4 markedly prolonged the viability of Sp2/0 cells, while the addition of PBS did not influence cell behavior (Fig, 2A and 2B). As expected, PBS supplementation did not prevent the increase in Gadd153 mRNA (Fig. 2C) or protein levels (Fig. 2D). However, supplementing the culture with L-glutamine on day 4 greatly reduced the induction of Gadd153 both at the mRNA (Fig. 2C) and protein levels (Fig. 2E) on culture day 6. Of note, the protein levels of Gadd153 remained very low up to the end of the L-glutamine-supplemented culture on day 10, even as the number of viable cells progressively decreased. For example, while the number of viable cells on day 5 in the control culture and on day 8 in the L-glutamine-supplemented sample is similar, Gadd153 proteins levels are high in the former and very low in the latter (Fig. 2D and 2E). These data indicate that, while Gadd153 expression correlates quite well with the health of the control Sp2/0 batch culture, its utility as a marker of cell stress is lost upon L-glutamine supplementation.

### Bcl-xL overexpression delays Gadd153 induction in batch cultures of Sp2/0 cells

**Figure 3:**
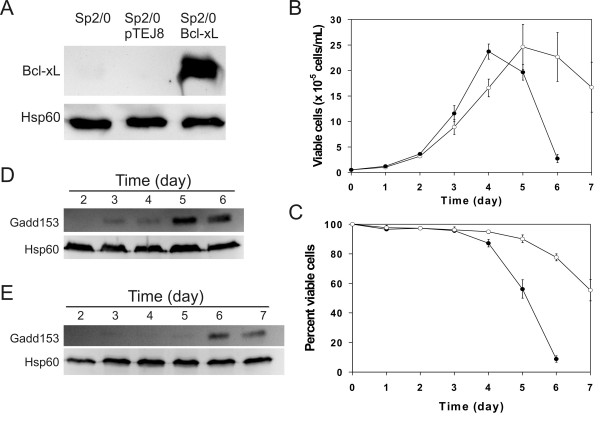
**Gadd153 expression in batch cultures of Bcl-xL-transfected Sp2/0 cells**. A) Immunoblot analysis of Bcl-xL expression in wild type Sp2/0, pTEJ8-Sp2/0 and Bcl-xL-Sp2/0 cells. B) Viable cell density. C) Percentage of viable cells in the culture. D) Immunoblot analysis of Gadd153 in batch cultures of the pTEJ8-transfected Sp2/0 cells. E) Immunoblot analysis of Gadd153 in batch cultures of the Bcl-xL-transfected Sp2/0 cells. Data in panels B and C are expressed as the average ± standard deviation of 3 independent experiments. Data in panels D and E are representative of 3 independent experiments. Open symbols: Bcl-xL-transfected cells. Closed symbols: vector-transfected control.

The viability of Sp2/0 cultures can also be improved by the ectopic expression of the anti-apoptotic protein Bcl-xL [16,18]. We therefore tested the effect of Bcl-xL expression on the induction of Gadd153 in Sp2/0 batch cultures. Western blot analysis revealed that the Bcl-xL-transfected cells expressed much higher levels of the protein than the wild type or vector-transfected controls (Fig, 3A). In agreement with our previously published data [16,18], the Bcl-xL overexpressing cells grew at a slower rate than the control, and their viability was also extended (Fig, 3B and 3C). Batch cultures of the vector-transfected control showed an increase in Gadd153 protein levels that was indistinguishable from the wild type Sp2/0 cells (compare Fig. 3D with Fig. 1B). Interestingly, the up-regulation of Gadd153 was delayed by one day in the Bcl-xL-transfected cells (Fig. 3E). As observed for the control cultures, the increased expression of Gadd153 in the Bcl-xL-Sp2/0 transfectants occurred as cell viability started to decline. Therefore, Gadd153 can be used to monitor the improvement of culture health attributable to ectopic Bcl-xL expression in Sp2/0 hybridomas.

### L-glutamine supplementation prevents the induction of Gadd153 in Bcl-xL-Sp2/0 cells in the absence of improvement in culture health

**Figure 4:**
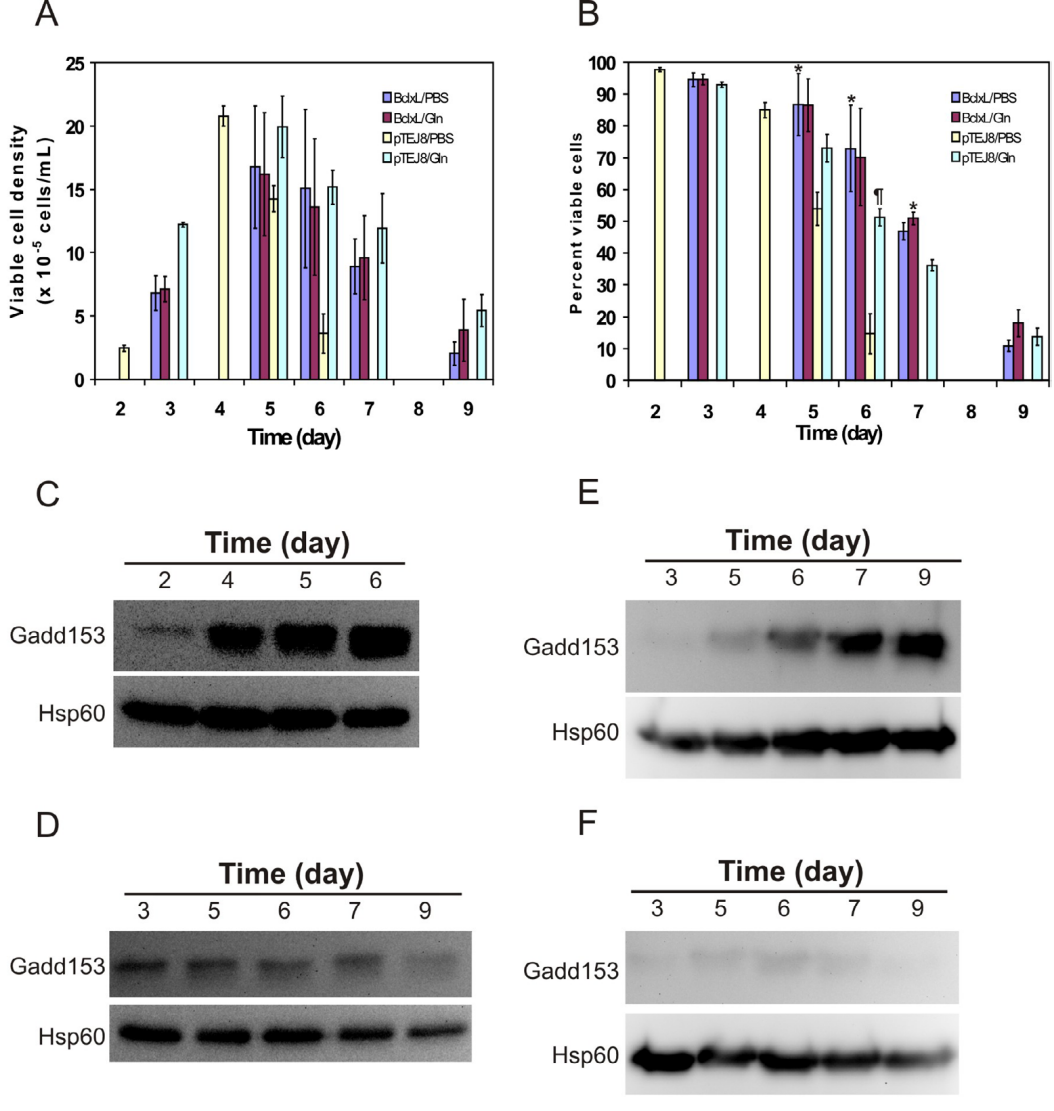
**Effect of L-glutamine supplementation on Gadd153 expression in batch cultures of Bcl-xL-transfected Sp2/0 cells**. Cells were processed for stationary batch cultures and supplemented with either PBS (panels C and E) or L-glutamine (Panels D and F) on culture day 4 (pTEJ8-Sp2/0) or day 5 (Bcl-xL-Sp2/0). A) Viable cell density. B) Percent viable cells. C and D) Immunoblot analysis of Gadd153 expression in pTEJ8-Sp2/0. E and F) Immunoblot analysis of Gadd153 in batch cultures of the Bcl-xL-transfected Sp2/0 cells. For panels A and B, data is the average ± standard deviation of three independent experiments. * = p < 0.01 vs pTEJ8 control. ¶= p < 0.05 vs PBS-supplemented control. Yellow bars: Sp2/0-pTEJ8 cells treated with PBS. Light blue bars: Sp2/0-pTEJ8 cells supplemented with L-glutamine. Dark blue bars: Sp2/0-BclxL cells supplemented with PBS. Dark red bars: Sp2/0-Bcl-xL cells treated with L-glutamine. Immunoblotting data are representative of 3 independent experiments.

We next investigated the expression of Gadd153 upon L-glutamine supplementation of Bcl-xL-Sp2/0 cultures. L-glutamine supplementation on culture day 4 lead to a significant increase in cell viability for the pTEJ8-Sp2/0 control cell line (Fig. 4A and 4B). Moreover, as observed for the wild type Sp2/0 cultures, L-glutamine addition severely blunted the increase in Gadd153 protein levels seen in the PBS-treated control (Fig, 4C and 4D). On the other hand, and in agreement with our previous observations [16], L-glutamine supplementation did not significantly increase cell viability in the Bcl-xL-transfected cells compared to the PBS-treated sample (Fig. 4A and 4B). However, L-glutamine addition did blunt the induction of Gadd153 protein in the Bcl-xL-Sp2/0 cells, while the culture treated with PBS showed an up-regulation of Gadd153 (Fig, 4E and 4F). Therefore, significant differences in Gadd153 levels were found in the L-glutamine- or PBS-treated Bcl-xl-Sp2/0 cultures even though cell viability was not different. Finally, and similar to our observations with the wild type Sp2/0 (Fig. 2), Gadd153 expression remained low in the L-glutamine-treated pTEJ8- and Bcl-xL-transfected cells, even as the number of viable cells progressively decreased (Fig, 4D and 4F). Thus, Gadd153 protein levels do not necessarily correlate with viability in cells transfected with Bcl-xL.

## Discussion

The optimization of mammalian cell culture processes would greatly benefit from the availability of a cellular marker that would 1) accurately reflect the health of the culture and 2) respond to strategies aimed at improving cell viability. Because its expression is induced by several stresses encountered during the large scale culture of mammalian cells, the pro-apoptotic transcription factor Gadd153 was recently proposed to be an indicator of apoptosis occurring in cell cultures [13,14].

To validate the suitability of Gadd153 as a stress marker for hybridomas, we studied its expression in Sp2/0 cells. Even though it does not secrete immunoglobulins [19], its high sensitivity to culture-related stresses and its low expression of the anti-apoptotic genes Bcl-2 and Bcl-xL make the Sp2/0 cell line a useful model for the study of optimization strategies aimed at improving cell viability. Monitoring the expression of Gadd153 in Sp2/0 cells grown under stationary batch culture conditions, we observed that both Gadd153 protein and RNA levels were markedly increased during the decline phase of the culture (Figs. 1 and 2). This is in line with previous studies [13,14] and confirm that, in batch culture, Gadd153 expression inversely correlates with Sp2/0 cell viability. These data also suggest that approaches based on the use of the Gadd153 promoter to induce the regulated expression of a gene of interest [20] should be applicable to the Sp2/0 cell model.

We also examined the regulation of Gadd153 under two conditions known to improve the behavior of the Sp2/0 cell line in batch culture: L-glutamine supplementation and the ectopic expression of Bcl-xL. We observed that Gadd153 mRNA and protein levels were significantly decreased in Sp2/0 cultures supplemented with L-glutamine (Fig. 2). A similar effect was also seen when Bcl-xL-overexpressing Sp2/0 cultures were supplemented with L-glutamine (Fig. 4). This response of Gadd153 is not surprising, considering that Gadd153 has been shown previously to be induced by amino acid starvation [8], that L-glutamine is the major limiting nutrient in Sp2/0 cells grown in stationary batch cultures [16], and that L-glutamine potently represses Gadd153 expression [21]. Thus, our results confirm those of Lengwehasatit and Dickson with the NS0 myeloma [13] and Murphy et al. in CHO cells [14] that Gadd153 expression is responsive to nutrient supplementation in a cell line of biotechnological interest.

Our data also revealed that the reduction in Gadd153 protein expression upon L-glutamine-supplementation persisted for the rest of the culture period, even as cell viability continued to decrease. This indicates that supplementing the culture with L-glutamine at the peak of Sp2/0 cell viability is sufficient to override further increases in Gadd153 protein levels, whatever the nature of the stresses leading to the loss of Sp2/0 cell viability. This phenomenon was not observed in other studies involving batch cultures because of the nature of the experimental design. Effectively, while Murphy et al. [14] limited their analysis of Gadd153 expression to 6 h after feeding, Lengwehasatit and Dickson [13] did a more thorough analysis of Gadd153 expression over several days of batch culture, but performed repeated nutrient supplementations during the culture period. Thus, our data indicate that while the expression of Gadd153 correlates well with the health of Sp2/0 cells in batch culture, it loses its utility as a stress marker upon supplementing the culture with L-glutamine. The cause of this lack of response of Gadd153 to the declining health of the L-glutamine-supplemented Sp2/0 cell culture remains unknown. An interesting possibility is that other stresses, such as the accumulation of lactate or ammonia, which are known to contribute to the reduction in hybridoma cell viability in stationary batch culture, interfere with the modulation of the expression of Gadd153.

We extended our studies to the use of Sp2/0 cells engineered to express high levels of the anti-apoptotic protein Bcl-xL. This manipulation was shown previously to significantly improve the behavior of Sp2/0 cells in batch culture [16,18]. Interestingly, the ectopic expression of Bcl-xL significantly prolonged Sp2/0 cell viability, which was accompanied by a delay in the increase in expression of Gadd153. However, L-glutamine-supplementation of the Bcl-xL-Sp2/0 cultures lead to a sustained decrease in Gadd153 protein expression in the absence of any benefit regarding cell viability. Because engineering mammalian cells to ectopically express anti-apoptotic genes is a major strategy for the improvement of cell lines of biotechnological importance [4], our results indicate that care should be taken when selecting Gadd153 protein expression as an indicator of the improvement in cell viability achieved with these approaches.

## Conclusion

The pro-apoptotic protein Gadd153 is acutely up-regulated by several stresses encountered during the large scale culture of mammalian cells. As such, its use as an indicator of cell stress can facilitate the optimization of cell cultures. However, our data highlight important limitations to the use of Gadd153 as a stress marker in mammalian batch cultures. Whether these limitations will be encountered in other cell lines or when other feeding mixtures or culture optimization strategies are used remains to be determined.

## Methods

### Reagents

Unless stated otherwise, all reagents were from Sigma-Aldrich (Oakville, ON). L-glutamine was prepared fresh as 200 mM stock solutions in phosphate buffered saline (PBS: 9.1 mM Na_2_HPO_4_, 1.7 mM NaH_2_PO_4_, 150 mM NaCl, pH 7.4) and adjusted to pH 7.2.

### Cell culture

Sp2/0 murine hybridoma cells were obtained from the American Type Culture Collection (Rockville, MD) and cultured in Iscove's Modified Dulbecco's Media (IMDM) supplemented with 5% Fetal Clone (Hyclone, Logan, UT), 100 U/mL penicillin, 100 μg/mL streptomycin and 4 mM L-glutamine (hereafter referred to as complete IMDM). The pTEJ8- and pTEJ8-Bcl-xL-transfected Sp2/0 cells were described previously [18] and were cultured in complete IMDM supplemented with 750 μg/mL G-418. Cell culture was performed at 37°C under a humidified atmosphere of 5% CO_2_.

Stationary batch culture and nutrient supplementation was done as described [16]. Briefly, exponentially growing Sp2/0 cells were centrifuged (500 × g, 10 min), washed once with warm PBS, and resuspended in complete IMDM at a concentration of 5 × 10^4 ^cells/mL. Samples were then taken at the indicated time intervals for the determination of cell viability using the trypan blue dye exclusion assay, and for total RNA and protein isolation. Culture supplementation with L-glutamine (4 mM final concentration) was done at the day where the maximal number of viable cells was observed in the controlled cultures, which is on culture day 4 for wild type Sp2/0 and pTEJ8-Sp2/0 cells and day 5 for the Bcl-xL-Sp2/0 transfectants [16].

### Reverse transcription-polymerase chain reaction (RT-PCR)

Total RNA isolation was performed as previously described [22]. Reverse transcription (RT) was carried out using conventional methods [23] in the presence of 1 μg RNA, 0.63 pmol/μL random hexamers (Canadian Life Technologies, Burlington ON), and 0.025 U/μL of Murine Moloney Leukemia Virus (M-MLV) reverse transcriptase (Canadian Life Technologies). For semi-quantitative polymerase chain reaction (PCR), cDNA dilutions were prepared so that all the samples yielded a comparable glyceraldehyde-3 phosphate dehydrogenase (GAPDH) signal upon amplification. PCR was done using a PTC-100HB thermocycler (MJ Research Inc., Waltham, MA) with the following cycling parameters: 5 cycles of touchdown PCR (95°C for 1 min, 65°C-1°C/cycle for 1 min, 68°C for 1 min) followed by 25 cycles of amplification (95°C for 1 min, 60°C for 1 min and 68°C for 1 min). The following primers pairs were used: GAPDH (Genebank: NM 008084): ^5'^ATGGTGAAGGTCGGTGTGAACGGA^3' ^(sense primer) and ^5'^TTACTCCTTGGAGGCCATGTAGGC^3' ^(antisense primer); Gadd153 (Genebank X67083): ^5'^ATGGCAGCTGAGTCCCTGCCT^3' ^(sense primer) and ^5'^TCACATGCTTGGCGCTGGCGC^3' ^(antisense primer). Samples were taken at cycles 21, 24, 27 and 30, and the amplicons were analyzed by agarose gel electrophoresis and ethidium bromide staining.

### Immunoblot analysis

Protein extracts were prepared using a urea-based lysis buffer (62.5 mM Tris-HCl, pH 6.8, 6 M urea, 10% glycerol, 2% SDS, 0.00125% bromophenol blue, 5% 2-mercaptoethanol), according to a procedure described by Shah *et al.*[24]. Proteins were separated by SDS-PAGE and transferred onto Hybond P membranes (GE Healthcare, Baie d'Urfé, QC). Transfer efficiency was routinely monitored by staining the membrane with Ponceau S. Immunoblotting was done by first blocking the membrane for 1 h at room temperature in Blotto (5% non fat dried milk in 0.02 M Tris-HCl, 0.14 M NaCl, 0.1% Tween 20, pH 7.6). The membrane was then incubated (1 h, room temperature) with the following primary antibodies (Santa Cruz Biotechnology Inc, Santa Cruz, CA) diluted in blotto: mouse monoclonal GADD153 antibody (clone B3, 1/200 dilution) and rabbit polyclonal Hsp60 antibody (1/2000 dilution). Detection of Hsp60 was performed to ensure equal protein loading of the wells. Detection by chemiluminescence was done by incubating the membrane for 1 h at room temperature with the appropriate secondary antibodies coupled to horseradish peroxidase (diluted 1/5000 in blotto) followed by the ChemiGlow reagent (Alpha Innotech, San Leandro, CA). Data acquisition was done with the Fluorchem 8000 Image Analysis System (Alpha Innotech).

### Statistical analysis

Statistical significance was determined using a one-way analysis of variance and Scheffe's post-hoc test.

## Authors' contributions

MM did the majority of the experiments of Figs. 1, 2, 3. KC did the experiments shown in Fig. 4. ERG conceived and coordinated the study, did the RT-PCR experiment and wrote the manuscript. All authors read and approved the final version of the manuscript.

## References

[B1] Barnes LM, Bentley CM, Dickson AJ (2000). Advances in animal cell recombinant protein production: the GS-NSO expression system. Cytotechnology.

[B2] Hu WS, Aunins JG (1997). Large-scale mammalian cell culture. Curr Opin Biotechnol.

[B3] Franek F (1995). Starvation-induced programmed death of hybridoma cells: prevention by amino acid mixtures. Biotechnology and Bioengineering.

[B4] Kuysterman D, Krampe B, Swiderek H, Al-Rubeai M (2007). Using cell engineering and omic tools for the improvement of cell culture processes. Cytotechnology.

[B5] Nissom PM, Sanny A, Kok YJ, Hiang YT, Chuah SH, Shing TK, Lee YY, Wong KT, Hu WS, Sim MY, Philp R (2006). Transcriptome and proteome profiling to understanding the biology of high productivity CHO cells. Mol Biotechnol.

[B6] Van Dyk DD, Misztal DR, Wilkins MR, Mackintosh JA, Poljak A, Varnai JC, Teber E, Walsh BJ, Gray PP (2003). Identification of cellular changes associated with increased production of human growth hormone in a recombinant Chinese hamster ovary cell line. Proteomics.

[B7] Luethy JD, Fargnoli J, Park JS, Fornace AJ, Holbrook NJ (1990). Isolation and characterization of the hamster gadd153 gene. Activation of promoter activity by agents that damage DNA. J Biol Chem.

[B8] Marten NW, Burke EJ, Hayden JM, Straus DS (1994). Effect of amino acid limitation on the expression of 19 genes in rat hepatoma cells. Faseb J.

[B9] Carlson SG, Fawcett TW, Bartlett JD, Bernier M, Holbrook NJ (1993). Regulation of the C/EBP-related gene GADD153 by glucose deprivation. Mol Cell Biol.

[B10] Wang XZ, Lawson B, Brewer JW, Zinszner H, Sanjay A, Mi LJ, Boorstein R, Kreibich G, Hendershot LM, Ron D (1996). Signals from the stressed endoplasmic reticulum induce C/EBP-homologous protein (CHOP/GADD153). Mol Cell Biol.

[B11] Kultz D, Madhany S, Burg MB (1998). Hyperosmolality causes growth arrest of murine kidney cells. Induction of GADD45 and GADD153 by osmosensing via stress-activated protein kinase 2. J Biol Chem.

[B12] Price BD, Calderwood SK (1992). GADD45 and GADD153 messenger RNA levels are increased during hypoxia and after exposure of cells to agents which elevate the levels of the glucose-regulated proteins. Cancer Res.

[B13] Lengwehasatit I, Dickson AJ (2002). Analysis of the role of GADD153 in the control of apoptosis in NS0 myeloma cells. Biotechnol Bioeng.

[B14] Murphy TC, Woods NR, Dickson AJ (2001). Expression of the transcription factor GADD153 is an indicator of apoptosis for recombinant chinese hamster ovary (CHO) cells. Biotechnol Bioeng.

[B15] Cudna RE, Dickson AJ (2006). Engineering responsiveness to cell culture stresses: growth arrest and DNA damage gene 153 (GADD153) and the unfolded protein response (UPR) in NS0 myeloma cells. Biotechnol Bioeng.

[B16] Charbonneau JR, Furtak T, Lefebvre J, Gauthier ER (2003). Bcl-xL expression interferes with the effects of L-glutamine supplementation on hybridoma cultures. Biotechnol Bioeng.

[B17] Gauthier ER, Piché L, Lemieux G, Lemieux R (1996). Role of bcl-xL in the control of apoptosis in murine myeloma cells. Cancer Research.

[B18] Charbonneau JR, Gauthier ER (2000). Prolongation of murine hybridoma cell survival in stationary batch culture by Bcl-xL expression. Cytotechnology.

[B19] Shulman M, Wilde CD, Kohler G (1978). A better cell line for making hybridomas secreting specific antibodies. Nature.

[B20] de Boer L, Gray PP, Sunstrom NA (2004). Enhanced productivity of G1 phase Chinese hamster ovary cells using the GADD153 promoter. Biotechnol Lett.

[B21] Huang Q, Lau SS, Monks TJ (1999). Induction of gadd153 mRNA by nutrient deprivation is overcome by glutamine. Biochem J.

[B22] Gauthier ER, Madison SD, Michel RN (1997). Rapid RNA isolation without the use of commercial kits: application to small tissue samples. Pflugers Arch.

[B23] Ausubel F, Brent R, Kingston RE, Moore DD, Seidman JG, Smith JA, Struhl K, Janssen K (1995). Current Protocols in Molecular Biology. John Wiley and Sons, Inc New York (NY).

[B24] Shah GM, Poirier D, Duchaine C, Brochu G, Desnoyers S, Lagueux J, Verreault A, Hoflack JC, Kirkland JB, Poirier GG (1995). Methods for biochemical study of poly(ADP-ribose) metabolism in vitro and in vivo. Anal Biochem.

